# The glucocorticoid dose-mortality nexus in pneumonia patients: unveiling the threshold effect

**DOI:** 10.3389/fphar.2024.1445979

**Published:** 2024-09-19

**Authors:** Saibin Wang, Qian Ye

**Affiliations:** ^1^ Department of Pulmonary and Critical Care Medicine, Jinhua Municipal Central Hospital, Jinhua, Zhejiang Province, China; ^2^ School of Medicine, Shaoxing University, Shaoxing, Zhejiang Province, China; ^3^ Department of Medical Records Quality Management, Jinhua Municipal Central Hospital, Jinhua, Zhejiang Province, China

**Keywords:** glucocorticoids, pneumonia, mortality, threshold effect, gender

## Abstract

**Background:**

The impact of glucocorticoid use on mortality risk in pneumonia patients remains unclear. This study aimed to investigate the relationship between the accumulated dose of glucocorticoids (ADG) and secondary pneumonia mortality risk among patients receiving oral or intravenous glucocorticoids.

**Methods:**

Data from the DRYAD database were analyzed, covering pneumonia patients from six academic hospitals over a 5-year period who had been administered oral or intravenous glucocorticoids. Piecewise linear regression and multivariate regression analysis were utilized to assess the association between ADG and mortality risk in pneumonia patients, while adjusting for potential confounders.

**Results:**

Among the 628 pneumonia patients included, the 30-day mortality rate was 23.1% and the 90-day mortality rate was 26.4%. In the high-dose glucocorticoid group (≥24 mg/day of methylprednisolone or an equivalent glucocorticoid within 30 days before admission), the 30-day and 90-day mortality rates were 31.2% and 35.9%, respectively. Piecewise linear regression analysis demonstrated a non-linear relationship between ADG and mortality risk in pneumonia patients. Multivariate regression analysis revealed a significantly lower mortality risk in patients receiving an ADG of 20–39 g methylprednisolone compared to those receiving lower (<20 g) or higher doses (≥40 g), after adjusting for potential confounding factors. Additionally, in the high-dose glucocorticoid group, surpassing the inflection point of 20 g of methylprednisolone raised the 30-day and 90-day mortality risks (adjusted odds ratio, 95% confidence interval: 1.16, 1.03–1.30 and 1.23, 1.07–1.42, respectively). Notably, this threshold effect was observed exclusively in male patients.

**Conclusion:**

This study provides evidence supporting a potential threshold effect between ADG and mortality risk in oral or intravenous glucocorticoid users with secondary pneumonia. Specifically, male patients receiving high-dose glucocorticoids should undergo close monitoring when the ADG of methylprednisolone exceeds 20 g, as it may be associated with an elevated risk of mortality.

## Introduction

Pulmonary infections commonly afflict immunocompromised patients undergoing glucocorticoid therapy (Agustí et al., 2003). Prolonged and high-dose glucocorticoid use can induce severe immunosuppression, heightening susceptibility to serious infections (Agustí et al., 2003; Guarnotta et al., 2021; Elsouri et al., 2023). Mortality rates soar, reaching up to 45% in rheumatic disease patients on glucocorticoids who develop pulmonary infections, escalating to a staggering 93% among those requiring mechanical ventilation (Agustí et al., 2003). Yet, the threshold for determining tolerably safe doses of glucocorticoid therapy remains elusive.

In evaluating dose-related major complications of glucocorticoids use, including infections, a multidisciplinary panel of experts from the European League Against Rheumatism, involving rheumatic disease patients, found that the risk of harm was minimal for most patients receiving long-term dosages of ≤5 mg prednisone equivalent per day. Conversely, at doses exceeding 10 mg/day, the risk of harm significantly increased. For dosages ranging between >5 and ≤10 mg/day, the risk of harm hinged on patient-specific characteristics, encompassing protective and risk factors (Strehl et al., 2016). Nonetheless, comprehensive evidence on the risk of harm associated with long-term or high-dose glucocorticoid therapy remains scant, with pertinent study findings often being either absent, contradictory, or carrying a high risk of bias (Soo et al., 2024; Peng et al., 2023; Sun et al., 2020; Tang et al., 2022). Addressing these knowledge gaps, our study endeavored to elucidate the specific correlation between cumulative glucocorticoid utilization and mortality risk among pneumonia patients undergoing glucocorticoids therapy. By delving into this intricate relationship, our aim is to furnish valuable insights into the judicious administration of glucocorticoid therapy in the management of pneumonia, particularly among immunosuppressed cohorts.

## Methods

### Study design and subjects

**FIGURE 1:**
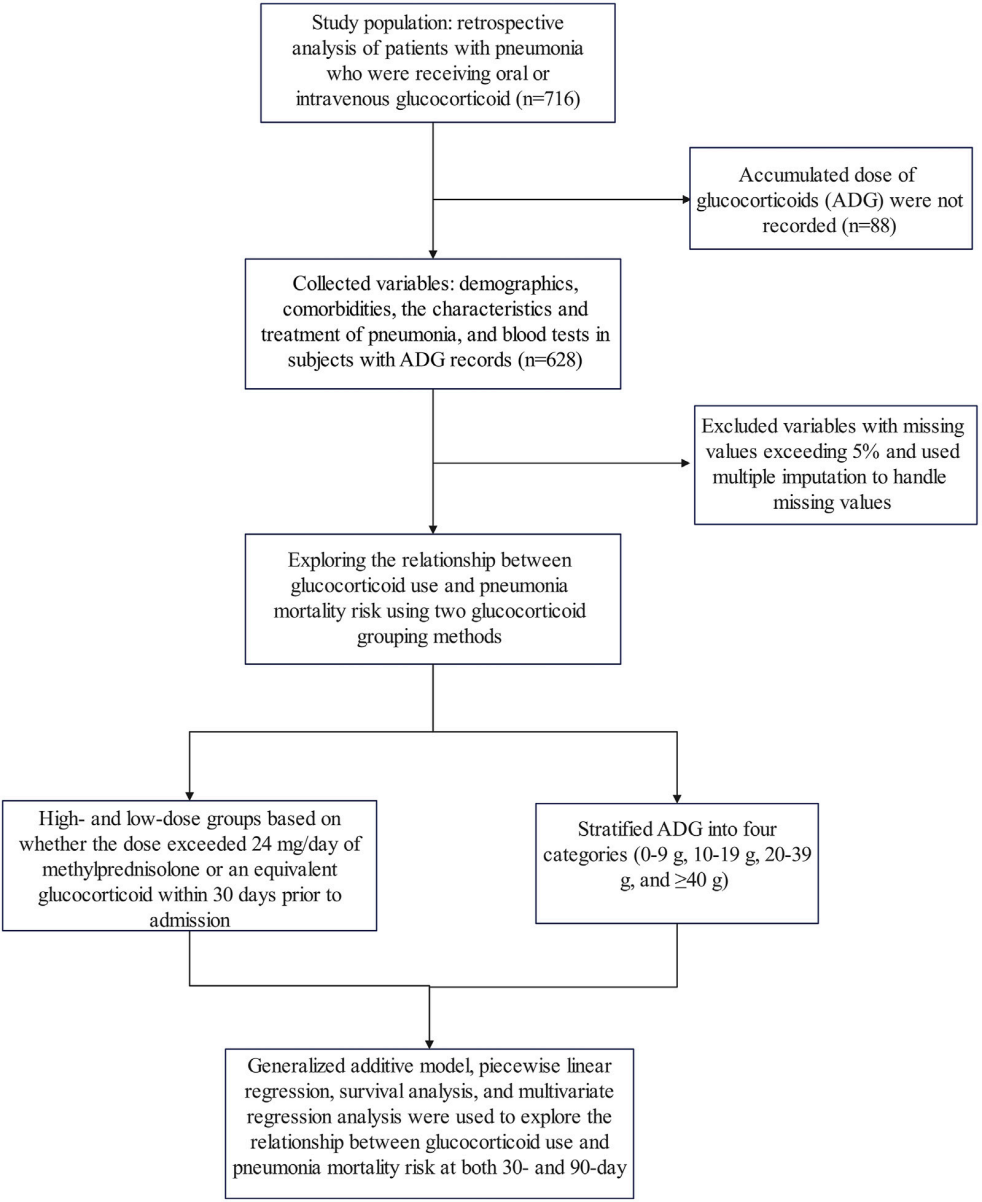
Study flow chart.

This study represents a secondary analysis of data obtained from the public database www.Datadryad.org, which allows unrestricted use of the data for research and educational purposes (Li et al., 2020a). The cohort consisted of 716 patients who received oral or intravenous glucocorticoid therapy with pneumonia and were recruited from six secondary and tertiary academic hospitals in China between January 2013 and December 2017 (Li et al., 2020b). Pneumonia diagnoses were established in accordance with the guidelines outlined by the American Thoracic Society and the Infectious Disease Society of America (Sousa et al., 2013; American Thoracic Society, Infectious Diseases Society of America, 2005). Patients included in the study met the following inclusion criteria: (1) receipt of oral or intravenous glucocorticoid treatment before admission; (2) diagnosis of pneumonia upon admission or during hospitalization; and (3) age of at least 16 years. Exclusion criteria encompassed: (1) diagnosis of non-infectious pulmonary conditions such as lung cancer, interstitial lung diseases unrelated to infection, pulmonary embolism, or heart failure; and (2) inability to provide consent for procedures (Li et al., 2020b). Furthermore, in this study, variables for which no accumulated dose of glucocorticoids (ADG) was recorded and those with missing values exceeding 5% were excluded from the analysis. The study flow chart is shown in Figure 1. The Ethics Committee of China-Japan Friendship Hospital (No. 2015-86) and all participating institutions granted approval for the study (Li et al., 2020b).

### Variables collection

The study encompassed patient demographics, including gender, age, smoking and alcoholism history. Comorbid diseases such as chronic obstructive pulmonary disease (COPD), asthma, bronchiectasis, idiopathic interstitial pneumonia, interstitial lung disease, hypertension, coronary heart disease (CHD), diabetes mellitus, nephrotic syndrome, chronic renal failure, cirrhosis, connective tissue disease (CTD), cerebrovascular disease, tumor, anemia, bone marrow transplantation, solid organ transplantation, leukemia, and radiation pneumonia were also recorded. Additionally, the characteristics and treatment of pneumonia, including community-acquired pneumonia (CAP) or hospital-acquired pneumonia (HAP), etiology determined from sputum and/or bronchoalveolar lavage samples, respiratory failure, CURB-65 score, pneumonia severity index, antibiotics, antiviral therapy, anti-*Aspergillus* treatment, use of extracorporeal membrane oxygenation, continuous veno-venous hemofiltration (CVVH), mechanical ventilation, vasoactive drugs, and other immunosuppressants were documented. Furthermore, blood tests were conducted to evaluate white blood cell count, neutrophil count, lymphocyte count, hemoglobin levels, platelet counts, alanine aminotransferase (ALT), aspartate aminotransferase, blood urea nitrogen (BUN), and serum creatinine levels. The variables included in this analysis are detailed in Table 1.

**TABLE 1:** Clinical characteristics of patients with pneumonia receiving oral or intravenous glucocorticoids.

Characteristics	30-day death		*P*-value	90-day death		*P*-value
	No (n = 483)	Yes (n = 145)		No (n = 462)	Yes (n = 166)	
Male, n (%)	247 (51.14)	76 (52.41)	0.788	235 (50.87)	88 (53.01)	0.635
Age >60 years, n (%)	157 (32.51)	43 (29.66)	0.518	146 (31.60)	54 (32.53)	0.826
Smoke, n (%)	114 (23.60)	35 (24.14)	0.131	108 (23.38)	41 (24.70)	0.237
Alcoholism, n (%)	35 (7.25)	14 (9.66)	0.343	34 (7.36)	15 (9.04)	0.49
Diseases, n (%)						
COPD	49 (10.14)	6 (4.14)	0.025	47 (10.17)	8 (4.82)	0.036
Asthma	12 (2.48)	2 (1.38)	0.429	12 (2.60)	2 (1.20)	0.297
Bronchiectasia	16 (3.31)	1 (0.69)	0.088	16 (3.46)	1 (0.60)	0.051
IIP	50 (10.35)	13 (8.97)	0.626	47 (10.17)	16 (9.64)	0.844
ILD	232 (48.03)	77 (53.10)	0.284	216 (46.75)	93 (56.02)	0.040
Hypertension	162 (33.54)	55 (37.93)	0.330	153 (33.12)	64 (38.55)	0.206
CHD	58 (12.01)	17 (11.72)	0.926	54 (11.69)	21 (12.65)	0.743
Diabetes mellitus	121 (25.05)	39 (26.90)	0.655	114 (24.68)	46 (27.71)	0.441
Nephrotic Syndrome	59 (12.22)	20 (13.79)	0.615	57 (12.34)	22 (13.25)	0.760
CRF	40 (8.28)	12 (8.28)	0.998	38 (8.23)	14 (8.43)	0.933
Cirrhosis	5 (1.04)	1 (0.69)	0.708	5 (1.08)	1 (0.60)	0.586
CTD	250 (51.76)	85 (58.62)	0.146	237 (51.30)	98 (59.04)	0.087
Cerebrovascular disease	40 (8.28)	6 (4.14)	0.093	38 (8.23)	8 (4.82)	0.149
Tumor	26 (5.38)	11 (7.59)	0.323	24 (5.19)	13 (7.83)	0.216
Anemia	43 (8.90)	21 (14.48)	0.051	38 (8.23)	26 (15.66)	0.007
Bone marrow transplantation	4 (0.83)	1 (0.69)	0.869	4 (0.87)	1 (0.60)	0.743
Solid organ transplantation	53 (10.97)	7 (4.83)	0.027	52 (11.26)	8 (4.82)	0.016
Leukemia	4 (0.83)	1 (0.69)	0.869	4 (0.87)	1 (0.60)	0.743
Radiation pneumonia	5 (1.04)	1 (0.69)	0.708	5 (1.08)	1 (0.60)	0.586
Lymphoma	10 (2.07)	3 (2.07)	0.999	9 (1.95)	4 (2.41)	0.720
Persistent lymphocytopenia	177 (36.65)	98 (67.59)	<0.001	166 (35.93)	109 (65.66)	<0.001
Pneumonia and treatment, n (%)						
CAP	436 (90.27)	125 (86.21)	0.165	419 (90.69)	142 (85.54)	0.065
Respiratory failure	189 (39.13)	141 (97.24)	<0.001	169 (36.58)	161 (96.99)	<0.001
CURB-65 score > 1	119 (24.64)	72 (49.66)	<0.001	108 (23.38)	83 (50.00)	<0.001
Pneumonia severity index	73.00 (55.50, 93.00)	95.00 (74.00, 122.00)	<0.001	72.00 (55.00, 92.00)	95.50 (75.00, 122.00)	<0.001
Etiology			0.015			0.003
*Pneumocystis*	28 (5.80)	10 (6.90)		27 (5.84)	11 (6.63)	
*Cytomegalovirus*	35 (7.25)	12 (8.28)		33 (7.14)	14 (8.43)	
*Pseudomonas*	9 (1.86)	1 (0.69)		9 (1.95)	1 (0.60)	
*Acinetobacter*	5 (1.04)	3 (2.07)		5 (1.08)	3 (1.81)	
*Aspergillus*	9 (1.86)	2 (1.38)		8 (1.73)	3 (1.81)	
*Klebsiella pneumonia*e	7 (1.45)	0 (0.00)		7 (1.52)	0 (0.00)	
Other pathogens^a^	70 (14.49)	7 (4.83)		68 (14.72)	9 (5.42)	
Mixed pathogens^b^ I	61 (12.63)	21 (14.48)		58 (12.55)	24 (14.46)	
Mixed pathogens II	115 (23.81)	50 (34.48)		105 (22.73)	60 (36.14)	
Mixed pathogens III	11 (2.28)	7 (4.83)		11 (2.38)	7 (4.22)	
Non-identified	133 (27.54)	32 (22.07)		131 (28.35)	34 (20.48)	
Antibiotics	319 (66.05)	112 (77.24)	0.011	302 (65.37)	129 (77.71)	0.003
Antiviral therapy	66 (13.66)	37 (25.52)	<0.001	59 (12.77)	44 (26.51)	<0.001
Anti-*Aspergillus*	172 (35.61)	92 (63.45)	<0.001	155 (33.55)	109 (65.66)	<0.001
ECMO	19 (3.93)	17 (11.72)	<0.001	16 (3.46)	20 (12.05)	<0.001
CVVH	22 (4.55)	43 (29.66)	<0.001	16 (3.46)	49 (29.52)	<0.001
Ventilation	121 (25.05)	120 (82.76)	<0.001	100 (21.65)	141 (84.94)	<0.001
Vasoactive drugs	34 (7.04)	83 (57.64)	<0.001	21 (4.55)	96 (58.18)	<0.001
Immunosuppressant	182 (37.68)	55 (37.93)	0.957	173 (37.45)	64 (38.55)	0.801
High-dose glucocorticoids#	163 (33.75)	74 (51.03)	<0.001	152 (32.90)	85 (51.20)	<0.001
Accumulated dose of glucocorticoids, methylprednisolone (g), median (IQR)	4.32 (2.16, 9.66)	2.88 (1.50, 4.80)	<0.001	4.32 (2.16, 10.06)	2.92 (1.50, 5.01)	<0.001
Blood test, median (IQR)						
White blood cell (×10^9^/L)	7.69 (5.64, 10.87)	10.04 (5.88, 13.01)	<0.001	7.61 (5.59, 10.77)	10.12 (6.33, 13.19)	<0.001
Neutrophils (×10^9^/L)	6.20 (4.16, 9.11)	8.34 (5.27, 11.78)	<0.001	6.12 (4.04, 8.93)	8.52 (5.37, 11.72)	<0.001
Lymphocyte (×10^9^/L)	0.95 (0.57, 1.46)	0.60 (0.36, 0.89)	<0.001	0.93 (0.57, 1.49)	0.64 (0.37, 1.01)	<0.001
Hemoglobin, g/L	113.00 (96.87, 129.00)	108.00 (92.00, 122.00)	0.036	114.00 (97.00, 129.00)	108.00 (91.25, 120.75)	0.009
Platelet counts (×10^9^/L)	193.00 (139.50, 256.50)	157.00 (102.00, 222.00)	<0.001	193.00 (140.00, 256.75)	158.00 (102.00, 227.75)	<0.001
AST, U/L	22.00 (16.00, 35.00)	36.90 (22.00, 66.00)	<0.001	22.00 (16.00, 34.00)	36.95 (21.25, 61.98)	<0.001
ALT, U/L	24.00 (15.00, 46.00)	29.00 (19.00, 57.70)	0.003	23.00 (15.00, 46.64)	28.00 (18.25, 54.95)	0.004
BUN, mmol/L	6.02 (4.45, 8.90)	8.21 (5.43, 13.85)	<0.001	6.00 (4.39, 8.80)	8.28 (5.55, 13.81)	<0.001
Serum creatinine, mmol/L	63.70 (49.80, 88.75)	70.50 (52.30, 122.30)	0.027	63.70 (50.20, 87.97)	70.65 (50.20, 118.35)	0.049

^a^ Other single pathogen identified including: *Influenza A virus, Influenza B virus,* Respiratory syncytial virus*, Herpes simplex virus type 1, Adenovirus, Humanrhinovirus, Mycobacterium tuberculosis, Haemophilus influenzae, Enterobacter cloacae, Burkholderia, Enterococcus, Stenotrophomonas, Escherichia coli, Comamonas acidovorans, and Legionella*.

^b^ Mixed pathogens were classified based on the presence or absence of the following pathogens: *Pneumocystis, Cytomegalovirus, Pseudomonas, Acinetobacter, Aspergillus, and Klebsiella pneumoniae*. Mixed pathogens I involved one of the listed pathogens, while mixed pathogens II, involved at least two, and mixed pathogens III, did not include any of the listed pathogens.

^#^ High-dose glucocorticoid use was defined as ≥24 mg/day of methylprednisolone or an equivalent glucocorticoid within 30 days before admission.

COPD, chronic obstructive pulmonary disease; IIP, idiopathic interstitial pneumonia; ILP, interstitial lung disease; ILD, interstitial lung disease; CHD, coronary heart disease; CRF, chronic renal failure; CTD, connective tissue disease; CAP, community-acquired pneumonia; ECMO, extracorporeal membrane oxygenation; CVVH, continuous venovenous hemofiltration; AST, aspartate aminotransferase; ALT, alanine aminotransferase; BUN, blood urea nitrogen.

In this study, we examined mortality risk among patients with secondary pneumonia receiving oral or intravenous glucocorticoid therapy by employing both the glucocorticoids dose stratification commonly used in previous research (Hoes et al., 2009; Buttgereit et al., 2002) and ADG stratification developed for this study. We categorized glucocorticoid use as follows: (1) High-dose glucocorticoid use was defined as ≥24 mg/day of methylprednisolone or an equivalent glucocorticoid within 30 days prior to admission, while low-dose glucocorticoid use was defined as <24 mg/day. (2) ADG was defined as the total amount of oral or intravenous glucocorticoids administered from the initiation of glucocorticoid therapy for underlying conditions until the diagnosis of pneumonia. Based on the thresholds identified in our analysis, we further stratified ADG into four categories (0–9 g, 10–19 g, 20–39 g, and ≥40 g) to explore their association with mortality risk.

### Statistical analysis

The baseline characteristics of the subjects were summarized in the study. Categorical variables were presented as counts (percentage), while continuous variables were expressed as median (interquartile range, IQR). Two-group comparisons between the deceased and surviving groups were conducted using unpaired *t*-tests or Wilcoxon rank-sum tests for continuous variables, and Pearson’s chi-squared tests or Fisher’s exact tests for categorical variables, as appropriate. Multiple imputation was employed to address missing values. To examine the association between ADG and the risk of pneumonia death, with adjustment for potential confounders, smoothing curve fitting via a generalized additive model was utilized. Additionally, an adjusted two-piecewise linear regression analysis, coupled with the log-likelihood ratio test, was employed to identify the threshold effect of ADG on the risk of pneumonia death. Survival analysis, including Kaplan-Meier analysis and log-rank test, was employed to compare differences between high and low glucocorticoid doses. Multivariate regression analysis was conducted to assess the independent relationship between ADG and the risk of pneumonia death, with adjustment for potential confounders. In this study, two criteria were employed to adjust for potential confounders: criteria I involved the use of a covariate-discrimination algorithm, whereby variables were included if their introduction into the basic model or removal from the complete model resulted in a change in the regression coefficient of ≥10%. Covariates meeting these criteria included neutrophils, COPD, CTD, ALT, BUN, CVVH, respiratory failure, and solid organ transplantation. Criteria II involved adjusting variables based on clinical considerations, including age, etiology, and the use of other immunosuppressants. All statistical analyses were performed using R software (version 3.5.1), and a P-value <0.05 was considered statistically significant.

## Results

The baseline data and characteristics of the pneumonia patients are detailed in Table 1. Among the 628 subjects who received oral or intravenous glucocorticoids and subsequently developed pneumonia, 323 (51.4%) were male, with 31.8% of patients aged over 60 years. The classification of pneumonia revealed that 561 (89.3%) individuals were CAP with 42.2% experiencing mixed pathogens. *Pneumocystis* and *Cytomegalovirus* were identified as the most common single-pathogen infections among the patients. In this study population, a total of 237 individuals (37.7%) underwent high-dose glucocorticoid therapy, with the median ADG (IQR) calculated at 3.8 (1.9–8.8) g. The median (IQR) duration of glucocorticoids use was 4 (2, 18) months, with an average duration of 22 months. The 30-day and 90-day mortality rates were recorded at 23.1% and 26.4%, respectively, reflecting the severity of the condition and the challenges associated with pneumonia management in this patient cohort.

**FIGURE 2:**
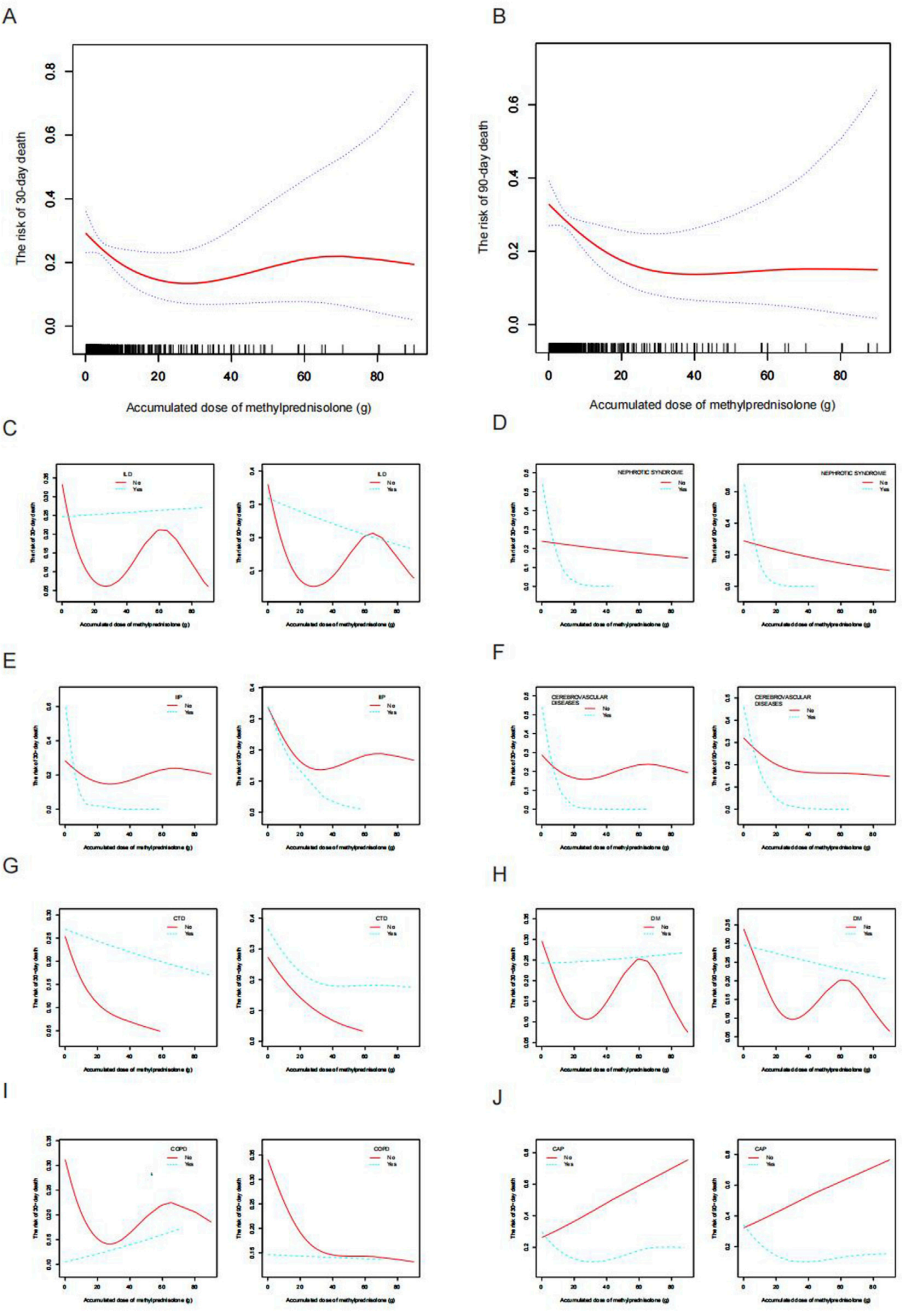
Smooth fitting curve illustrating the association between ADG (methylprednisolone) and the mortality risk among patients receiving glucocorticoid with secondary pneumonia. **(A)** The risk of 30-day death and **(B)** The risk of 90-day death. The ADG and risk of pneumonia death in the presence of **(C)** ILD, **(D)** Nephrotic syndrome, **(E)** IIP, **(F)** Cerebrovascular disease, **(G)** CTD, **(H)** Diabetes mellitus, **(I)** COPD, and **(J)** CAP. Adjustments were made for age, etiology, other immunosuppressants, neutrophils, COPD, CTD, ALT, BUN, CVVH, respiratory failure, and solid organ transplantation, except where the variable was analyzed. ADG, accumulated dose of glucocorticoids; ILD, interstitial lung disease; IIP, interstitial lung disease; CTD, connective tissue disease; COPD, chronic obstructive pulmonary disease; CAP, community-acquired pneumonia; ALT, alanine aminotransferase; BUN, blood urea nitrogen; CVVH, continuous venovenous hemofiltration.

A non-linear relationship between ADG and pneumonia mortality at both 30-day and 90-day endpoints was observed after adjustment for potential confounders (age, etiology, other immunosuppressants use, neutrophils, COPD, CTD, ALT, BUN, CVVH, respiratory failure, and solid organ transplantation), as illustrated in Figures 2A, B. This phenomenon was also evident in subgroup analyses based on different underlying diseases and pneumonia types (Figures 2C–J). Further stratifying ADG into four subgroups (0–9 g, 10–19 g, 20–39 g, and ≥40 g), multivariate regression analysis revealed that compared to the 20–39 g group, the 0–9 g, 10–19 g, and ≥40 g groups had adjusted odds ratios (aOR) and 95% confidence interval (CI) for 30-day and 90-day pneumonia mortality of 5.36 (1.36, 21.03), 5.59 (1.10, 28.48), 6.64 (1.18, 37.32), and 8.78 (2.04, 37.88), 6.88 (1.24, 38.09), 6.55 (1.07, 40.05), respectively. Moreover, the P-values for trend tests were all greater than 0.05, further confirming the presence of a non-linear relationship between ADG dose and pneumonia mortality (Table 2).

**TABLE 2:** Multivariate regression analysis of accumulated dose of methylprednisolone with the risk of mortality in patients with pneumonia.

Exposure	Model I: Non-adjusted		Model II: Adjusted^a^
30-day mortality, OR (95% CI) *P*-value			
Accumulated dose of methylprednisolone (g)			
20–39	1.0	1.0	
0–9	3.98 (1.41, 11.28) 0.0093	5.36 (1.36, 21.03) 0.0161	
10–19	2.03 (0.59, 7.04) 0.2627	5.59 (1.10, 28.48) 0.0385	
≥40	5.04 (1.39, 18.21) 0.0137	6.64 (1.18, 37.32) 0.0315	
*P* for trend	0.1027	0.3516	
90-day mortality, OR (95% CI) *P*-value			
20–39	1.0	1.0	
0–9	4.90 (1.73, 13.85) 0.0027	8.78 (2.04, 37.88) 0.0036	
10–19	2.30 (0.68, 7.85) 0.1819	6.88 (1.24, 38.09) 0.0271	
≥40	5.04 (1.39, 18.21) 0.0137	6.55 (1.07, 40.05) 0.0418	
*P* for trend	0.0188	0.0576	

^a^ Adjust for: age, etiology, other immunosuppressants (methotrexate, cyclosporine, cyclophosphamide, tacrolimus, sirolimus and azathioprine), neutrophils, COPD, CTD, ALT, BUN, CVVH, respiratory failure, solid organ transplantation.

COPD, chronic obstructive pulmonary disease; CTD, connective tissue disease; ALT, alanine aminotransferase; BUN, blood urea nitrogen; CVVH, continuous venovenous hemofiltration.

**FIGURE 3:**
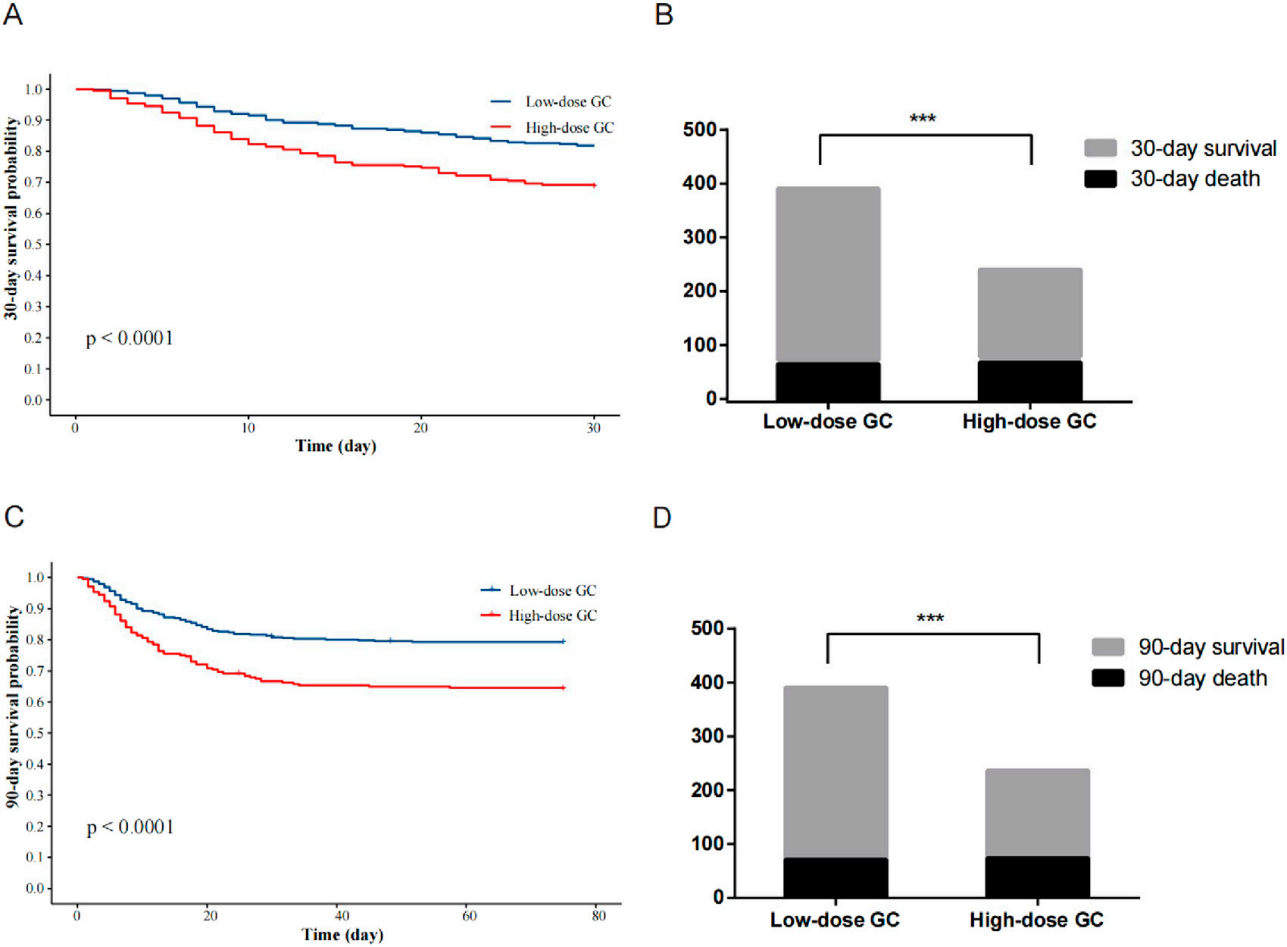
Kaplan-Meier analysis and comparison of mortality between high- and low-dose glucocorticoid groups. **(A)** 30-day survival curve analysis. **(B)** Comparison of 30-day mortality. **(C)** 90-day survival curve analysis. **(D)** Comparison of 90-day mortality. GC, glucocorticoid. ***P < 0.001.

Patients with pneumonia who received high-dose glucocorticoids therapy exhibited higher 30-day and 90-day mortality rates compared to those in the low-dose glucocorticoid group, with rates of 31.2% versus 18.2% and 35.9% versus 20.7%, respectively, demonstrating statistically significant differences between the two groups (Figure 3, *P* < 0.001). In contrast to patients receiving low-dose glucocorticoids, those in the high-dose group exhibited a lower proportion of patients aged over 60 years, a higher percentage with CTD, and a greater incidence of respiratory failure (67.51%). These patients had shorter durations of oral or intravenous glucocorticoid use, resulting in lower ADG compared to the low-dose glucocorticoid group. Moreover, a smaller proportion of patients in the high-dose group received concurrent treatment with other immunosuppressants (methotrexate, cyclosporine, cyclophosphamide, tacrolimus, sirolimus, and azathioprine) compared to the low-dose group (55.70% vs. 66.24%, *P* < 0.05). Regarding pathogen profiles, the incidence of *Pneumocystis* was higher among patients in the high-dose glucocorticoid group compared to those in the low-dose group. In terms of treatment, a larger proportion of patients (54.01%) in the high-dose glucocorticoid group required mechanical ventilation. Additional clinical characteristics of patients in the high-dose group can be found in Supplementary Table S1. In the high-dose glucocorticoid group, males accounted for 54.9% (130/237). Among comorbidities, males had a higher proportion of CHD compared to females, while the proportion of CTD was lower in males. Male patients also exhibited a higher percentage of pneumonia with CURB-65 score >1 and higher pneumonia severity index (*P* < 0.05). Additionally, in terms of blood tests, males showed significantly higher levels of renal function markers (serum creatinine and BUN) compared to females. Additional demographic characteristics and differences between genders are shown in Supplementary Table S2.

**FIGURE 4:**
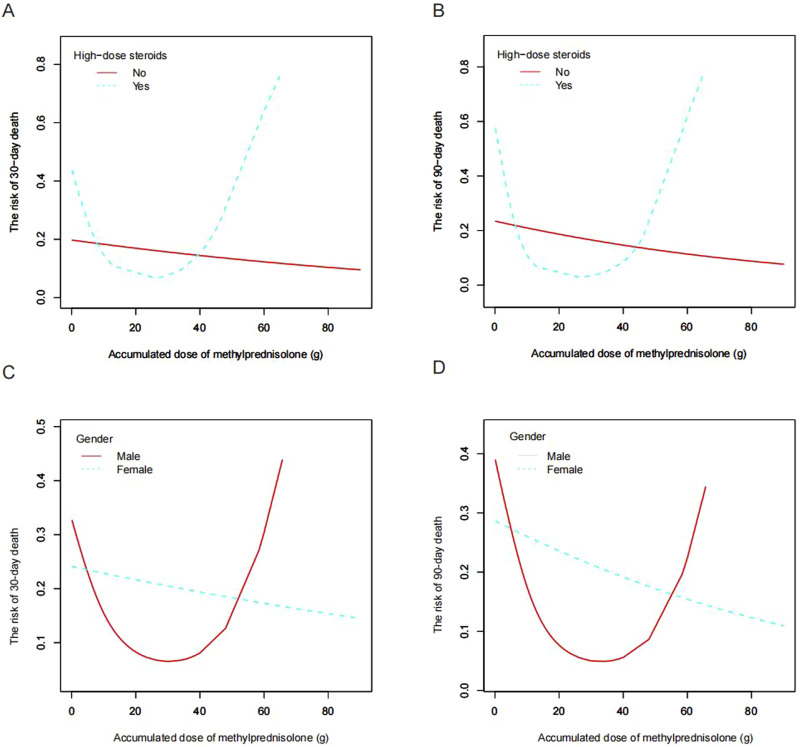
Smooth fitting curves depict a U-shaped correlation between ADG (methylprednisolone) and the mortality risk among pneumonia patients in the high-dose glucocorticoid population (depicted by the blue dotted line) for **(A)** 30-day mortality risk and **(B)** 90-day mortality risk. Notably, this U-shaped correlation pattern is exclusively observed in male patients (illustrated by the red line) for **(C)** 30-day mortality and **(D)** 90-day mortality. Adjustments were made for age, etiology, other immunosuppressants, neutrophils, COPD, CTD, ALT, BUN, CVVH, respiratory failure, and solid organ transplantation. ADG, accumulated dose of glucocorticoids; COPD, chronic obstructive pulmonary disease; CTD, connective tissue disease; ALT, alanine aminotransferase; BUN, blood urea nitrogen; CVVH, continuous venovenous hemofiltration.

In light of the non-linear relationship between ADG and pneumonia mortality risk, we conducted an in-depth analysis to explore the potential threshold effect between these two variables. Our findings revealed that the inflection point for the ADG associated with a 30-day mortality risk in the overall study population was 21.9 g, while for the 90-day mortality risk, it was 23.4 g (Table 3, *P* < 0.05). Subsequent subgroup analyses indicated that this threshold effect was only present in the high-dose glucocorticoid group and not in the low-dose glucocorticoid group (Table 3; Figures 4A, B). Specifically, within the high-dose glucocorticoid group, the risk of 30-day pneumonia mortality increased when the ADG exceeded 20 g (aOR 1.16, 95% CI 1.03–1.30, *P* = 0.0173), and there was a similar increase in the risk of 90-day mortality (aOR 1.23, 95% CI 1.07–1.42, *P* = 0.0040). This signifies that for each additional ADG unit beyond 20 g, the risk of death at 30 and 90 days increased by 16% and 23%, respectively (Table 3). These findings underscore the importance of considering the ADG as a critical factor in evaluating pneumonia mortality risk, particularly in patients receiving high-dose glucocorticoid therapy.

**TABLE 3:** Threshold effect of accumulated dose of methylprednisolone on the risk of mortality in patients with pneumonia in adjusted two-piecewise linear regression.

30-day mortality, adjusted OR (95% CI) *P*-value			
Accumulated dose of methylprednisolone (g)	Low-dose group	High-dose group^a^	Total
Inflection point (K)	21.9	20	21.9
<K-segment effect	0.95 (0.89, 1.01) 0.1109	0.81 (0.67, 0.98) 0.0307	0.94 (0.89, 0.99) 0.0234
>K-segment effect	1.02 (0.98, 1.06) 0.4548	1.16 (1.03, 1.30) 0.0173	1.02 (0.99, 1.06) 0.2120
The difference in effect between the two K-segments	1.07 (0.98, 1.17) 0.1514	1.42 (1.06, 1.91) 0.0185	1.09 (1.00, 1.18) 0.0382
Predicted value of equation at K	−2.36 (−3.13, −1.59)	−4.78 (−7.19, −2.38)	−2.66 (−3.41, −1.92)
Log-likelihood ratio	0.149	0.010	0.035
90-day mortality, adjusted OR (95% CI) *P*-value			
Inflection point (K)	2.4	20	23.4
<K-segment effect	1.57 (0.88, 2.81) 0.1296	0.68 (0.54, 0.86) 0.0014	0.94 (0.89, 0.99) 0.0130
>K-segment effect	0.98 (0.96, 1.00) 0.1030	1.23 (1.07, 1.42) 0.0040	1.02 (0.98, 1.06) 0.3916
The difference in effect between the two K-segments	0.62 (0.35, 1.13) 0.1182	1.81 (1.26, 2.59) 0.0013	1.09 (1.00, 1.18) 0.0532
Predicted value of equation at K	−1.27 (−1.61, −0.92)	−5.39 (−7.85, −2.93)	−2.51 (−3.23, −1.78)
Log-likelihood ratio	0.114	<0.001	0.041

^a^ High-dose glucocorticoid use was defined as ≥24 mg/day of methylprednisolone or an equivalent glucocorticoid within 30 days before admission.

Adjust for: age, etiology, other immunosuppressants (methotrexate, cyclosporine, cyclophosphamide, tacrolimus, sirolimus and azathioprine), neutrophils, COPD, CTD, ALT, BUN, CVVH, respiratory failure, solid organ transplantation.

COPD, chronic obstructive pulmonary disease; CTD, connective tissue disease; ALT, alanine aminotransferase; BUN, blood urea nitrogen; CVVH, continuous venovenous hemofiltration.

Furthermore, a notable observation emerged regarding a gender-specific difference in the threshold effect. Specifically, within the high-dose glucocorticoid group, the threshold effect of ADG on the risk of pneumonia mortality was evident solely among male patients, with no such effect observed among female patients (Figures 4C, D).

## Discussion

The current investigation unveiled a non-linear association between ADG and pneumonia mortality risk at both 30- and 90-day. Specifically, among male individuals receiving high doses of glucocorticoids, there was a discernible increase in pneumonia-related mortality risk when ADG exceeded 20 g.

The intricate interplay between glucocorticoid usage and pneumonia remains a captivating subject of inquiry. On one hand, a plethora of studies have unequivocally affirmed the therapeutic efficacy of glucocorticoids in the management of pneumonia, particularly in cases characterized by rapid progression or severe manifestations such as acute respiratory distress syndrome (Pinzón et al., 2021; Chan et al., 2020). The timely administration of glucocorticoids at appropriate dosages has been shown to ameliorate gas exchange, diminish the duration of recovery, reduce the necessity for escalation to intensive care, and in some instances, mitigate mortality rates (Soo et al., 2024; Pinzón et al., 2021; Souza et al., 2022). On the contrary, prolonged and high-dose administration of glucocorticoids can induce a state of immunosuppression in the body, rendering individuals more susceptible to pulmonary infections. This relationship has been substantiated by previous studies, which have frequently documented instances where long-term and high-dose glucocorticoid usage predisposes individuals to an increased risk of developing pneumonia (Guarnotta et al., 2021; Elsouri et al., 2023; Tang et al., 2022). Glucocorticoids, as endogenous hormones, exert regulatory effects on nearly all cells within the body (Quatrini and Ugolini, 2021). Additionally, long-term use of glucocorticoids may result in lasting adverse effects that significantly impact a patient’s quality of life, persisting even after discontinuation of therapy (Seguro et al., 2013). Evidence suggests an increased risk of infections, osteoporosis, osteonecrosis, cardiovascular disease, and cancer among individuals with a history of glucocorticoid use (Seguro et al., 2013). Given the intricate and wide-ranging influence of glucocorticoids on immune function, it is imperative to carefully calibrate the dosage of glucocorticoid administration to strike a delicate balance between eradicating pathogens and safeguarding against excessive inflammation and immunopathology in infectious diseases.

This study has revealed a non-linear association between the ADG and the risk of mortality in pneumonia patients undergoing glucocorticoid therapy. Specifically, the analysis demonstrated a U-shaped relationship, wherein an incremental increase in ADG corresponded to a gradual reduction in the risk of pneumonia-related mortality, followed by an upturn in risk at higher doses. This pattern mirrors observations in previous reports, wherein moderate glucocorticoid dosing facilitated pneumonia recovery and diminishes the risk of mortality (Pinzón et al., 2021; Chan et al., 2020), while excessive glucocorticoid doses were associated with heightened susceptibility to pneumonia and increased mortality risk (Agustí et al., 2003; Guarnotta et al., 2021; Zhao et al., 2022). In our study, we observed that the mortality was higher at an ADG of <20 g methylprednisolone compared to the 20–39 g dose group. This finding may be related to the significantly higher rate of respiratory failure in the <20 g group (54.48%) compared to the 20–39 g group (31.37%). Although there is no previous research directly linking ADG with pneumonia mortality risk, existing literature suggests that prolonged and appropriate glucocorticoid therapy can reduce systemic inflammation, improve lung and extrapulmonary organ function, shorten mechanical ventilation duration, and decrease ICU stay, potentially leading to lower mortality rates (Annane et al., 2017; Villar et al., 2020; Meduri et al., 2009). In this study, we also categorized glucocorticoid use into high-dose and low-dose groups using a commonly employed stratification method based on previous research (Hoes et al., 2009; Buttgereit et al., 2002). A novel finding from our study is the identification of a distinct turning point within the U-shaped relationship observed in pneumonia patients receiving glucocorticoids, occurring at approximately 20 g of ADG. Notably, this phenomenon was observed exclusively in individuals receiving high-dose glucocorticoid therapy. This finding carries significant implications, suggesting that the ADG should be carefully considered and meticulously monitored in patients undergoing high-dose glucocorticoid treatment.

Another interesting finding in this study pertains to the gender-specific variations in the relationship between the ADG and the risk of pneumonia-related mortality. Specifically, while the U-shaped relationship and threshold effect were evident among male patients, they were not observed in female patients. Gender disparities in immune function, neurosteroid levels, stress responses, and pharmacological responses have been well-documented in the context of disease progression (Hodes et al., 2024). Notably, significant gender differences have been observed in the severity of symptoms and mortality rates associated with pneumonia, including COVID-19, which may be attributed to variations in immune responses (Spini et al., 2021). Furthermore, gender variations in the response to glucocorticoid therapy have been reported (Lucafò et al., 2021; Czock et al., 2005). Czock et al. proposed that females may have distinct drug metabolism dynamics compared to males, noting potentially higher clearance rates of methylprednisolone in females (Czock et al., 2005). Such gender-specific variations in methylprednisolone clearance could partially elucidate the observed gender disparities in the association between ADG and mortality risk in pneumonia identified in our study. However, a more in-depth mechanistic explanation may require further research. Our findings underscore the importance of recognizing and addressing gender-specific factors in glucocorticoid therapy. It is imperative to incorporate gender-tailored strategies into glucocorticoid therapy protocols for pneumonia.

## Limitations

Our study is subject to several noteworthy limitations that warrant acknowledgment. Firstly, the retrospective design inherently exposes the study to confounding factors and selection biases, despite efforts to adjust for major confounders. For instance, some pathogens were identified through sputum cultures, potentially not fully representing pulmonary infection pathogens. Moreover, the delayed identification of certain pathogens until at least 48 h post-admission increases the likelihood of nosocomial infections, thereby possibly inaccurately distinguishing between CAP and HAP in some cases. Additionally, variables reflecting patients’ immune status, such as immunoglobulin levels and albumin, were not controlled for in this study due to their absence or high levels of missing data in the original dataset. In our study, over half of the patients were receiving other concurrent immunosuppressive treatments. While we adjusted for the presence or absence of such treatments in our analysis, we acknowledge that we did not account for the use of different categories of immunosuppressants. This limitation should be considered when interpreting our results. Secondly, the assessment of ADG only accounted for intravenous or oral glucocorticoid dosages, disregarding the doses of inhaled glucocorticoids. Patients with conditions such as asthma and COPD often receive concurrent inhaled glucocorticoid therapy, potentially leading to an underestimation of the risk of pneumonia mortality associated with ADG in this subset of patients. Thirdly, Owing to insufficient data regarding essential variables in our dataset, validated glucocorticoid toxicity scores such as the glucocorticoid toxicity index or its abbreviated form, the glucocorticoid toxicity index-metabolic domains (Patel et al., 2023), could not be included in this analysis. Additionally, our investigation exclusively focused on methylprednisolone without exploring other glucocorticoids, thereby limiting the generalizability of our findings concerning the relationship between various glucocorticoids and mortality risk. Despite these acknowledged limitations, our study represents a pioneering effort in elucidating the specific relationship between ADG and the risk of pneumonia-related mortality in populations receiving glucocorticoid therapy.

## Conclusion

In summary, our investigation reveals a non-linear association between cumulative glucocorticoid usage and the risk of pneumonia-related mortality in populations receiving glucocorticoids. Particularly, the ADG exceeding 20 g may emerge as an independent risk factor for pneumonia-related mortality in male pneumonia patients. These findings underscore the pivotal role of ADG in assessing and managing the risk of pneumonia-related mortality. Through elucidating this relationship, our study furnishes clinicians with valuable insights to actively assess and alleviate the risk of pneumonia-related mortality, thereby enhancing the refinement of therapeutic approaches and the optimization of patient care within this clinical realm.

## Data Availability

The original contributions presented in the study are included in the article/Supplementary Material, further inquiries can be directed to the corresponding author.
