# Supplementary material for: The glucocorticoid dose-mortality nexus in pneumonia patients: unveiling the threshold effect
Source: Front Pharmacol. 2024 Sep 19;15:1445979. doi: 10.3389/fphar.2024.1445979 (PMC11447404; doi:10.3389/fphar.2024.1445979)
Supplement: Supplementary file 1 [file Table1.DOCX]

Supplementary Table 1 Clinical characteristics of patients receiving different doses of oral or intravenous glucocorticoids

| High-dose glucocorticoids | No (n=391) | Yes (n=237) | Standardize diff. | P-value |
| --- | --- | --- | --- | --- |
| Male, n(%) | 193 (49.36%) | 130 (54.85%) | 0.11 (-0.05, 0.27) | 0.182 |
| Age >60 years, n (%) | 213 (54.48%) | 98 (41.35%) | 0.27 (0.10, 0.43) | 0.01 |
| Smoker, n (%) | 96 (24.55%) | 53 (22.36%) | 0.05 (-0.11, 0.21) | 0.822 |
| Alcoholism, n (%) | 25 (6.39%) | 24 (10.13%) | 0.14 (-0.03, 0.30) | 0.091 |
| COPD, n (%) | 39 (9.97%) | 16 (6.75%) | 0.12 (-0.04, 0.28) | 0.166 |
| Asthma, n (%) | 9 (2.30%) | 5 (2.11%) | 0.01 (-0.15, 0.17) | 0.874 |
| Bronchiectasia, n (%) | 15 (3.84%) | 2 (0.84%) | 0.20 (0.04, 0.36) | 0.025 |
| IIP, n (%) | 40 (10.23%) | 23 (9.70%) | 0.02 (-0.14, 0.18) | 0.832 |
| ILD, n (%) | 183 (46.80%) | 126 (53.16%) | 0.13 (-0.03, 0.29) | 0.122 |
| Hypertension, n (%) | 150 (38.36%) | 67 (28.27%) | 0.22 (0.05, 0.38) | 0.010 |
| CHD, n (%) | 53 (13.55%) | 22 (9.28%) | 0.13 (-0.03, 0.30) | 0.110 |
| Diabetes mellitus, n (%) | 101 (25.83%) | 59 (24.89%) | 0.02 (-0.14, 0.18) | 0.794 |
| Nephrotic Syndrome, n (%) | 51 (13.04%) | 28 (11.81%) | 0.04 (-0.12, 0.20) | 0.653 |
| CFR, n (%) | 38 (9.72%) | 14 (5.91%) | 0.14 (-0.02, 0.30) | 0.093 |
| Cirrhosis, n (%) | 5 (1.28%) | 1 (0.42%) | 0.09 (-0.07, 0.25) | 0.417 |
| CTD, n (%) | 195 (49.87%) | 140 (59.07%) | 0.19 (0.02, 0.35) | 0.025 |
| Cerebrovascular disease, n (%) | 34 (8.70%) | 12 (5.06%) | 0.14 (-0.02, 0.31) | 0.090 |
| Tumor, n (%) | 23 (5.88%) | 14 (5.91%) | 0.00 (-0.16, 0.16) | 0.990 |
| Anemia, n (%) | 40 (10.23%) | 24 (10.13%) | 0.00 (-0.16, 0.16) | 0.967 |
| Bone marrow transplantation, n (%) | 4 (1.02%) | 1 (0.42%) | 0.07 (-0.09, 0.23) | 0.655 |
| Solid organ transplantation, n (%) | 57 (14.58%) | 3 (1.27%) | 0.51 (0.34, 0.67) | <0.001 |
| Radiation pneumonia, n (%) | 4 (1.02%) | 2 (0.84%) | 0.02 (-0.14, 0.18) | 1.000 |
| Lymphoma, n (%) | 4 (1.02%) | 9 (3.80%) | 0.18 (0.02, 0.34) | 0.018 |
| Persistent lymphocytopenia, n (%) | 162 (41.43%) | 113 (47.68%) | 0.13 (-0.04, 0.29) | 0.126 |
| CAP, n (%) | 347 (88.75%) | 214 (90.30%) | 0.05 (-0.11, 0.21) | 0.542 |
| Respiratory failure, n (%) | 170 (43.48%) | 160 (67.51%) | 0.50 (0.33, 0.66) | <0.001 |
| CURB-65 score >1, n (%) | 124 (31.71%) | 67 (28.27%) | 0.08 (-0.09, 0.24) | 0.363 |
| Pneumonia severity index | 78.00 (60.50-100.00) | 76.00 (57.00-105.00) | 0.01 (-0.15, 0.17) | 0.919 |
| Etiology, n (%) |  |  | 0.53 (0.37, 0.70) | <0.001 |
| Non-identified | 127 (32.48%) | 38 (16.03%) |  |  |
| Pneumocystis | 16 (4.09%) | 22 (9.28%) |  |  |
| Cytomegalovirus | 25 (6.39%) | 22 (9.28%) |  |  |
| Pseudomonas | 6 (1.53%) | 4 (1.69%) |  |  |
| Acinetobacter | 5 (1.28%) | 3 (1.27%) |  |  |
| Aspergillus | 4 (1.02%) | 7 (2.95%) |  |  |
| Klebsiella pneumoniae | 5 (1.28%) | 2 (0.84%) |  |  |
| Other pathogens* | 55 (14.07%) | 22 (9.28%) |  |  |
| Mixed pathogens** I | 51 (13.04%) | 31 (13.08%) |  |  |
| Mixed pathogens II | 84 (21.48%) | 81 (34.18%) |  |  |
| Mixed pathogens III | 13 (3.32%) | 5 (2.11%) |  |  |
| Antibiotics, n (%) | 263 (67.26%) | 168 (70.89%) | 0.08 (-0.08, 0.24) | 0.343 |
| Antiviral therapy, n (%) | 65 (16.62%) | 38 (16.03%) | 0.02 (-0.15, 0.18) | 0.846 |
| Anti-*Aspergillus*, n (%) | 151 (38.62%) | 113 (47.68%) | 0.18 (0.02, 0.35) | 0.026 |
| ECMO, n (%) | 20 (5.12%) | 16 (6.75%) | 0.07 (-0.09, 0.23) | 0.393 |
| CVVH, n (%) | 37 (9.46%) | 28 (11.81%) | 0.08 (-0.09, 0.24) | 0.348 |
| Ventilation, n (%) | 113 (28.90%) | 128 (54.01%) | 0.53 (0.36, 0.69) | <0.001 |
| Vasoactive drugs, n (%) | 66 (16.92%) | 51 (21.52%) | 0.12 (-0.04, 0.28) | 0.152 |
| Immunosuppressant, n (%) | 259 (66.24%) | 132 (55.70%) | 0.22 (0.06, 0.38) | 0.008 |
| ADG (methylprednisolone) (g), median (IQR) | 5.10 (1.92-13.14) | 3.00 (2.00-4.80) | 0.44 (0.27, 0.60) | <0.001 |
| Duration of ADG, (month), median (IQR) | 10.00 (3.30-32.00) | 2.00 (1.00-3.00) | 0.62 (0.46, 0.79) | <0.001 |
| 30-day mortality, n (%) | 71 (18.16%) | 74 (31.22%) | 0.31 (0.14, 0.47) | <0.001 |
| 90-day mortality, n (%) | 81 (20.72%) | 85 (35.86%) | 0.34 (0.18, 0.50) | <0.001 |
| White blood cell (×10^9^/L) | 7.69 (5.54-11.10) | 8.90 (6.18-11.76) | 0.14 (-0.02, 0.30) | 0.010 |
| Neutrophils (×10^9^/L) | 6.11 (3.94-9.39) | 7.13 (4.77-10.75) | 0.21 (0.05, 0.38) | <0.001 |
| Lymphocyte (×10^9^/L) | 0.91 (0.57-1.48) | 0.72 (0.41-1.23) | 0.27 (0.11, 0.43) | <0.001 |
| Hemoglobin, g/L | 110.00 (93.00-126.00) | 115.00 (99.00-129.00) | 0.18 (0.02, 0.34) | 0.013 |
| Platelet counts (×10^9^/L) | 186.00 (136.00-251.50) | 185.00 (122.00-250.00) | 0.07 (-0.09, 0.23) | 0.494 |
| AST, U/L | 21.00 (15.55-35.00) | 30.00 (20.00-52.00) | 0.20 (0.04, 0.36) | <0.001 |
| ALT, U/L | 21.00 (14.00-37.05) | 34.00 (20.00-67.80) | 0.32 (0.16, 0.49) | <0.001 |
| BUN, mmol/L | 6.29 (4.45-10.57) | 6.28 (4.82-9.41) | 0.03 (-0.13, 0.19) | 0.814 |
| Serum creatinine, mmol/L | 66.00 (52.35-100.25) | 61.60 (46.30-88.50) | 0.16 (0.00, 0.33) | 0.021 |

*Other single pathogen identified including: *Influenza A virus, Influenza B virus, Respiratory syncytial virus, Herpes simplex virus type 1, Adenovirus, Humanrhinovirus, Mycobacterium tuberculosis, Haemophilus influenzae, Enterobacter cloacae, Burkholderia, Enterococcus, Stenotrophomonas, Escherichia coli, Comamonas acidovorans, and Legionella.*

**Mixed pathogens were classified based on the presence or absence of the following pathogens: *Pneumocystis, Cytomegalovirus, Pseudomonas, Acinetobacter, Aspergillus, and Klebsiella pneumoniae.* Mixed pathogens I involved one of the listed pathogens, while mixed pathogens II involved at least two, and mixed pathogens III did not include any of the listed pathogens.

^#^High-dose steroid use was defined as ≥24 mg/day of methylprednisolone or an equivalent glucocorticoid within 30 days before admission.

COPD, chronic obstructive pulmonary disease; IIP, Idiopathic interstitial pneumonia; ILP, interstitial lung disease; ILD, interstitial lung disease; CHD, coronary heart disease; CRF, chronic renal failure; CTD, connective tissue disease; CAP, community-acquired pneumonia; ECMO, extracorporeal membrane oxygenation; CVVH, continuous venovenous hemofiltration; ADG, accumulated dose of glucocorticoids; AST, aspartate aminotransferase; ALT, alanine aminotransferase; BUN, blood urea nitrogen.

Supplementary Table 2 Clinical characteristics of patients receiving high-dose glucocorticoids among different genders

| Gender | Male (n=130) | Female (n=107) | Standardize diff. | P-value |
| --- | --- | --- | --- | --- |
| Age >60 years, n (%) | 64 (49.23%) | 34 (31.78%) | 0.36 (0.10, 0.62) | 0.007 |
| Smoker, n (%) | 51 (39.23%) | 2 (1.87%) | 1.14 (0.86, 1.41) | <0.001 |
| Alcoholism, n (%) | 21 (16.15%) | 3 (2.80%) | 0.47 (0.21, 0.73) | <0.001 |
| COPD, n (%) | 12 (9.23%) | 4 (3.74%) | 0.22 (-0.03, 0.48) | 0.094 |
| Asthma, n (%) | 2 (1.54%) | 3 (2.80%) | 0.09 (-0.17, 0.34) | 0.660 |
| Bronchiectasia, n (%) | 0 (0.00%) | 2 (1.87%) | 0.20 (-0.06, 0.45) | 0.203 |
| IIP, n (%) | 17 (13.08%) | 6 (5.61%) | 0.26 (0.00, 0.52) | 0.053 |
| ILD, n (%) | 64 (49.23%) | 62 (57.94%) | 0.18 (-0.08, 0.43) | 0.181 |
| Hypertension, n (%) | 40 (30.77%) | 27 (25.23%) | 0.12 (-0.13, 0.38) | 0.346 |
| CHD, n (%) | 17 (13.08%) | 5 (4.67%) | 0.30 (0.04, 0.56) | 0.027 |
| Diabetes mellitus, n (%) | 36 (27.69%) | 23 (21.50%) | 0.14 (-0.11, 0.40) | 0.272 |
| Nephrotic Syndrome, n (%) | 18 (13.85%) | 10 (9.35%) | 0.14 (-0.12, 0.40) | 0.285 |
| CFR, n (%) | 10 (7.69%) | 4 (3.74%) | 0.17 (-0.09, 0.43) | 0.199 |
| Cirrhosis, n (%) | 0 (0.00%) | 1 (0.93%) | 0.14 (-0.12, 0.39) | 0.451 |
| CTD, n (%) | 64 (49.23%) | 76 (71.03%) | 0.46 (0.20, 0.72) | <0.001 |
| Cerebrovascular disease, n (%) | 6 (4.62%) | 6 (5.61%) | 0.05 (-0.21, 0.30) | 0.729 |
| Tumor, n (%) | 11 (8.46%) | 3 (2.80%) | 0.25 (-0.01, 0.50) | 0.066 |
| Anemia, n (%) | 13 (10.00%) | 11 (10.28%) | 0.01 (-0.25, 0.27) | 0.943 |
| Bone marrow transplantation, n (%) | 0 (0.00%) | 1 (0.93%) | 0.14 (-0.12, 0.39) | 0.451 |
| Solid organ transplantation, n (%) | 3 (2.31%) | 0 (0.00%) | 0.22 (-0.04, 0.47) | 0.254 |
| Radiation pneumonia, n (%) | 2 (1.54%) | 0 (0.00%) | 0.18 (-0.08, 0.43) | 0.503 |
| Lymphoma, n (%) | 6 (4.62%) | 3 (2.80%) | 0.10 (-0.16, 0.35) | 0.518 |
| Persistent lymphocytopenia, n (%) | 62 (47.69%) | 51 (47.66%) | 0.00 (-0.26, 0.26) | 0.996 |
| CAP, n (%) | 120 (92.31%) | 94 (87.85%) | 0.15 (-0.11, 0.41) | 0.249 |
| Respiratory failure, n (%) | 94 (72.31%) | 66 (61.68%) | 0.23 (-0.03, 0.48) | 0.082 |
| CURB-65 score >1, n (%) | 44 (33.85%) | 23 (21.50%) | 0.28 (0.02, 0.54) | 0.036 |
| Pneumonia severity index | 86.00 (66.25-114.50) | 67.00 (48.00-93.00) | 0.49 (0.23, 0.75) | <0.001 |
| Etiology, n (%) |  |  | 0.52 (0.26, 0.78) | 0.128 |
| Non-identified | 14 (10.77%) | 24 (22.43%) |  |  |
| Pneumocystis | 7 (5.38%) | 15 (14.02%) |  |  |
| Cytomegalovirus | 14 (10.77%) | 8 (7.48%) |  |  |
| Pseudomonas | 3 (2.31%) | 1 (0.93%) |  |  |
| Acinetobacter | 2 (1.54%) | 1 (0.93%) |  |  |
| Aspergillus | 5 (3.85%) | 2 (1.87%) |  |  |
| Klebsiella pneumoniae | 1 (0.77%) | 1 (0.93%) |  |  |
| Other pathogens* | 12 (9.23%) | 10 (9.35%) |  |  |
| Mixed pathogens** I | 19 (14.62%) | 12 (11.21%) |  |  |
| Mixed pathogens II | 51 (39.23%) | 30 (28.04%) |  |  |
| Mixed pathogens III | 2 (1.54%) | 3 (2.80%) |  |  |
| Antibiotics, n (%) | 101 (77.69%) | 67 (62.62%) | 0.33 (0.08, 0.59) | 0.011 |
| Antiviral therapy, n (%) | 30 (23.08%) | 8 (7.48%) | 0.44 (0.19, 0.70) | 0.001 |
| Anti-*Aspergillus*, n (%) | 66 (50.77%) | 47 (43.93%) | 0.14 (-0.12, 0.39) | 0.294 |
| ECMO, n (%) | 13 (10.00%) | 3 (2.80%) | 0.30 (0.04, 0.55) | 0.028 |
| CVVH, n (%) | 15 (11.54%) | 13 (12.15%) | 0.02 (-0.24, 0.27) | 0.885 |
| Ventilation, n (%) | 73 (56.15%) | 55 (51.40%) | 0.10 (-0.16, 0.35) | 0.465 |
| Vasoactive drugs, n (%) | 34 (26.15%) | 17 (15.89%) | 0.25 (-0.00, 0.51) | 0.056 |
| Immunosuppressant, n (%) | 62 (47.69%) | 70 (65.42%) | 0.36 (0.11, 0.62) | 0.006 |
| ADG (methylprednisolone) (g), median (IQR) | 2.88 (1.49-4.23) | 3.50 (2.40-5.65) | 0.25 (-0.00, 0.51) | 0.003 |
| Duration of ADG, (month), median (IQR) | 2.00 (1.00-3.00) | 3.00 (1.05-4.00) | 0.20 (-0.06, 0.45) | 0.001 |
| 30-day mortality, n (%) | 40 (30.77%) | 34 (31.78%) | 0.02 (-0.23, 0.28) | 0.868 |
| 90-day mortality, n (%) | 48 (36.92%) | 37 (34.58%) | 0.05 (-0.21, 0.30) | 0.708 |
| White blood cell (×10^9^/L) | 9.07 (6.45-11.72) | 8.55 (6.14-11.93) | 0.01 (-0.25, 0.26) | 0.829 |
| Neutrophils (×10^9^/L) | 7.20 (5.20-10.77) | 7.06 (4.71-10.68) | 0.01 (-0.25, 0.26) | 0.798 |
| Lymphocyte (×10^9^/L) | 0.72 (0.41-1.33) | 0.70 (0.42-1.10) | 0.02 (-0.23, 0.28) | 0.689 |
| Hemoglobin, g/L | 118.00 (99.25-130.00) | 112.00 (99.00-127.00) | 0.17 (-0.09, 0.43) | 0.290 |
| Platelet counts (×10^9^/L) | 164.00 (114.00-226.75) | 197.00 (137.50-261.50) | 0.33 (0.07, 0.59) | 0.012 |
| AST, U/L | 30.00 (19.00-51.25) | 30.00 (21.00-51.50) | 0.08 (-0.18, 0.34) | 0.768 |
| ALT, U/L | 36.00 (22.25-71.75) | 29.00 (16.00-58.50) | 0.08 (-0.18, 0.33) | 0.057 |
| BUN, mmol/L | 7.13 (5.29-10.72) | 5.77 (4.55-8.10) | 0.24 (-0.02, 0.49) | 0.001 |
| Serum creatinine, mmol/L | 71.45 (52.45-98.97) | 52.00 (42.40-73.45) | 0.32 (0.07, 0.58) | <0.001 |

*Other single pathogen identified including: *Influenza A virus, Influenza B virus, Respiratory syncytial virus, Herpes simplex virus type 1, Adenovirus, Humanrhinovirus, Mycobacterium tuberculosis, Haemophilus influenzae, Enterobacter cloacae, Burkholderia, Enterococcus, Stenotrophomonas, Escherichia coli, Comamonas acidovorans, and Legionella.*

**Mixed pathogens were classified based on the presence or absence of the following pathogens: *Pneumocystis, Cytomegalovirus, Pseudomonas, Acinetobacter, Aspergillus, and Klebsiella pneumoniae.* Mixed pathogens I involved one of the listed pathogens, while mixed pathogens II involved at least two, and mixed pathogens III did not include any of the listed pathogens.

^#^High-dose steroid use was defined as ≥24 mg/day of methylprednisolone or an equivalent glucocorticoid within 30 days before admission.

COPD, chronic obstructive pulmonary disease; IIP, Idiopathic interstitial pneumonia; ILP, interstitial lung disease; ILD, interstitial lung disease; CHD, coronary heart disease; CRF, chronic renal failure; CTD, connective tissue disease; CAP, community-acquired pneumonia; ECMO, extracorporeal membrane oxygenation; CVVH, continuous venovenous hemofiltration; ADG, accumulated dose of glucocorticoids; AST, aspartate aminotransferase; ALT, alanine aminotransferase; BUN, blood urea nitrogen.
